# Dietary Supplementation of* Hericium erinaceus* Increases Mossy Fiber-CA3 Hippocampal Neurotransmission and Recognition Memory in Wild-Type Mice

**DOI:** 10.1155/2017/3864340

**Published:** 2017-01-01

**Authors:** Federico Brandalise, Valentina Cesaroni, Andrej Gregori, Margherita Repetti, Chiara Romano, Germano Orrù, Laura Botta, Carolina Girometta, Maria Lidia Guglielminetti, Elena Savino, Paola Rossi

**Affiliations:** ^1^Brain Research Institute, University of Zurich, Winterthurerstrasse 190, 8057 Zurich, Switzerland; ^2^Department of Biology and Biotechnology (DBB) “L. Spallanzani”, University of Pavia, Via Ferrata 1, 27100 Pavia, Italy; ^3^MycoMedica d.o.o., Podkoren 72, 4280 Kranjska Gora, Slovenia; ^4^O. B. L. Department of Surgical Sciences, V. Ospedale 54, University of Cagliari, 09124 Cagliari, Italy; ^5^Department of Earth and Environmental Science (DSTA), University of Pavia, Via S. Epifanio 14, 27100 Pavia, Italy; ^6^Miconet s.r.l, Academic Spin-Off of the University of Pavia, Via Moruzzi 13, 27100 Pavia, Italy

## Abstract

*Hericium erinaceus* (Bull.) Pers. is a medicinal mushroom capable of inducing a large number of modulatory effects on human physiology ranging from the strengthening of the immune system to the improvement of cognitive functions. In mice, dietary supplementation with* H. erinaceus* prevents the impairment of spatial short-term and visual recognition memory in an Alzheimer model. Intriguingly other neurobiological effects have recently been reported like the effect on neurite outgrowth and differentiation in PC12 cells. Until now no investigations have been conducted to assess the impact of this dietary supplementation on brain function in healthy subjects. Therefore, we have faced the problem by considering the effect on cognitive skills and on hippocampal neurotransmission in wild-type mice. In wild-type mice the oral supplementation with* H. erinaceus* induces, in behaviour test, a significant improvement in the recognition memory and, in hippocampal slices, an increase in spontaneous and evoked excitatory synaptic current in mossy fiber-CA3 synapse. In conclusion, we have produced a series of findings in support of the concept that* H. erinaceus *induces a boost effect onto neuronal functions also in nonpathological conditions.

## 1. Introduction

*Hericium erinaceus* (Bull.) Pers. (also known as Yamabushitake, Lion's Mane, or Satyr's beard) is a mushroom that grows on both living and dead broadleaf trees. It has been used for centuries as food source and herbal medicine in several Asian countries. However, over the last 10 years, the potential nootropic capabilities of* H. erinaceus* in neurodegenerative diseases have attracted considerable attention [1].

In mice, dietary supplementation with* H. erinaceus* prevents the impairment of spatial short-term and visual recognition memory induced by amyloid *β*(25–35) peptide [2]. Recently, Hazekawa et al. [3] described the neuroprotective effects of* H. erinaceus* dietary supplementation in mice subjected to middle cerebral artery occlusion. Furthermore, scores on the cognitive function scale improved after oral administration of* H. erinaceus *in patients suffering from mild cognitive impairment [4]. Finally, in humans, reduction of depression and of anxiety following 4 weeks of* H. erinaceus* intake was described in 30 females by means of a questionnaire investigation for psychometric measures [5].

Most of the effects induced by* H. erinaceus* have been correlated with an increase in the NGF production. NGF is a small secreted protein that acts as a neurotrophin that plays pivotal roles in neuronal survival in the adult mammalian brain and modulates forms of structural and functional plasticity, like the neurogenesis and memory (for a review see [6]).

What is the link between NGF and* H. erinaceus *dietary supplementation? A study conducted by Mori et al. 2008 [7] has shown that the extract of the fruiting body of* H. erinaceus* presents a 5-fold increase in the level of expression of NGF mRNAs in isolated human astrocytoma cells with respect to the control. Similar effect was also described by others [8, 9]. Interestingly, NGF effect was detected in the hippocampus but not in the cortex, thus suggesting an essential role of NGF in hippocampal learning and memory [10]. Furthermore, the presence of a persistent neurogenesis has recently been discovered in a specific region of the hippocampus, the dentate gyrus; it involves the main neuronal type present there, the granule cells (for a review see [10]).

Despite an extensive investigation of the preventing action of* H. erinaceus *in cognitive pathological conditions, to date, no studies have investigated the effects of dietary supplementation with* H. erinaceus *in healthy mice. To approach this point a group of wild-type mice were fed with a dextrin dietary supplementation and another one with* H. erinaceus *dietary supplementation and we investigated the effect on behaviour and on the neuronal network involved in memory skills. We therefore performed in vivo and in vitro experiments to assess novelty exploration and recognition memory by a battery of spontaneous behavioural tests and the effect on hippocampal mossy fiber to CA3 pyramidal cells synapses in wild-type mice after two months of dietary supplementation with* H. erinaceus*.

The* H. erinaceus* supplemented mice were able to perform better in a novel object recognition test. They were also more adventurous in exploring a novel environment. Electrophysiology recording in the mossy fiber-CA3 region suggested that there was a higher amount of neurotransmitter release from mossy fiber to CA3. This conclusion was supported by higher frequency and higher amplitude of spontaneous excitatory activities and lower number of stimulation failures and decreased pair-pulse ratio of evoked activities in Hr-fed mice.

## 2. Materials and Methods

### 2.1. Subjects

Blind experiments were carried out in wild-type mice (strain C57BL-6J). To avoid any potential differences related to the oestrous cycle in females, only males were selected. One-month-old wild-type male mice were divided into two groups: control mice received 2 months of a diet containing 5% dextrin dietary supplementation (dx mice), and* Hericum erinaceus *treated mice (Hr mice) received two months of a diet containing 5% “Micotherapy Hericium” supplement (corresponding to 0.025 g/g body weight). Water was provided* ad libitum* for both groups.

After 2 months of dietary supplementation in vivo experiments were performed in dx and Hr mice. At the end of behavioural test session, the same animals were maintained with the same diet and about 1 week later were used for patch-clamp experiments.

### 2.2. Fungal Supplementation

The supplement “Micotherapy Hericium” was provided by A. V. D Reform s.r.l. (Noceto, Parma, Italy). The supplement contains mycelium and fruiting body extract of* Hericium erinaceus* in a ratio 4/1 (Table 1).* H. erinaceus* (Her. Erin. strain) culture was obtained from the fungal culture bank of MycoMedica d.o.o., Slovenia, and was cultivated in the dark on PDA (Potato Dextrose Agar; Difco, USA) at 24°C. After 20 days, cultures were transferred onto lignocellulosic substrates and further incubated for 60 days at 24°C. After incubation, fruiting bodies and fungal biomass were harvested.

Fruiting bodies extractions were performed for three hours using water and ethanol as solvents in a 1 : 15 extraction ratio (w/v). The remaining extracted liquid as well as mycelium was dried under vacuum at 70°C and −0.9 bar and further milled by using a UPZ mill (Hosokawa Alpine Aktiengesellschaft, Augsburg, Germany) to obtain particles mostly smaller than 100 *μ*m.

The polysaccharide content of* H. erinaceus * fruiting bodies extract and of* H. erinaceus* mycelium, contained in “Micotherapy Hericium” supplement, was determined using *β*-Glucan Assay Kit (Megazyme, Ireland) and expressed as total (*α* plus *β*) glucan content (Tables 2 and 3).

All experiments were carried out according to the guidelines laid down by the institution's animal welfare committee, the Ethics Committee of Pavia University.

### 2.3. Apparatus and Procedures for Behavioural Test

Motor activity was quantified by means of a SMART video tracking system (2 Biological Instruments, Besozzo, Varese, Italy) and a Sony CCD colour video camera (PAL).

#### 2.3.1. Emergence Test

To assess approach and exploratory behaviour in rodents, we performed the emergence test, which is a variant of the open-field test that was designed to reduce anxiety by providing a safe enclosure within the open field. This test has been used to test anxiety-like behaviour in mice [11–13]. The free exploration test entails housing animals in a compartment prior to giving the animal a free choice between a novel compartment and a familiar one [14]. In our experimental conditions, the mouse is situated in a familiar environment (a cage 33 cm long, 15 cm wide, and 13 cm high) with a hole in one side (5 cm long and 4 cm wide) through which it can emerge in a larger arena (90 cm long and 60 cm wide) with a laminated floor but without walls. While the primary measure of anxiety-like behaviour is taken to be the latency to emerge into the novel arena, auxiliary markers of anxiety-like behaviour may include the number of emergences and the time spent out of the cage.

#### 2.3.2. Novel Object Recognition Task

The novel object recognition task (NOR) is used to test novelty exploration behaviour and recognition memory in rodents [15, 16]. The task consists of three phases: habituation, familiarization, and the test. In the habituation phase, for the first two days each mouse is given 10 minutes to freely explore the open-field arena in the absence of objects, after which it is removed from the arena and placed in the holding cage. On the third day, during the familiarization phase, each mouse is placed into the open-field arena and left free to explore two identical objects for 5 minutes. After the retention phase has elapsed (15 minutes), the mouse is put back into the open box where it is exposed both to a familiar object, identical to the one previously encountered in the familiarization phase, and to a novel object with a different size and shape. Approaches are defined as nose entries within 2 cm far from the object.

In both behavioural tests, the apparatus was wiped clean with water and dried after every trial.

### 2.4. Behavioural Test Analysis

In the emergence test, the software collected the following:

1. The number of times a mouse completely emerged from the cage with all four limbs.
2. The amount of time a mouse spent exploring the large environment outside the cage.
3. The latency before the first exit from the cage.

In the NOR task, the software collected the following:

1. The number of approaches.
2. Total duration of approaches.
3. Average duration of an approach.
4. Latency of the first approach.
5. Total latency between approaches.
6. Average latency between approaches.

### 2.5. Hippocampal Slices Preparation

250 *μ*m thick hippocampal sagittal slices were prepared from mice treated with either placebo or* H. erinaceus *by oral administration for two months. Briefly, mice were anesthetized by isofluorane inhalation (Aldrich, Milwaukee, WI, USA) before they were decapitated (the experimental procedure was approved by the Ethical Committee of the University of Pavia; Regulation of the Italian Ministry of Sanity, number 68/97-A). Artificial cerebrospinal fluid (ACSF) solution for slice cutting and recovery contained (in mM) the following: NaCl 120, KCl 2, MgSO_4_ 1.2, NaHCO_3_ 26, KH_2_PO_4_ 1.2, CaCl_2_ 2, and glucose 11. This solution was equilibrated with 95% O_2_ and 5% CO_2_, pH 7.4. Slices were maintained at room temperature before being transferred to the recording chamber (1.5 ml) mounted on the stage of an upright microscope (OLYMPUS BX51WI, Japan). The preparation was then superfused at a rate of 2 ml/min with Krebs' solution and maintained at 30°C by a feedback Peltier device (TC-324B, Warner Instr. Corp., Hamden, CT, USA).

### 2.6. Electrophysiological Data Collection and Analysis

Patch-clamp whole-cell recordings were performed in CA3 pyramidal neurons. CA3-CA3 and mossy fiber-CA3 synapses were studied; spontaneous and evoked postsynaptic current were recorded in placebo and treated mice. No more than two neurons have been recorded from the same mice.

Whole-cell patch-clamp recordings were made from the soma of visually identified CA3 pyramidal neurons and membrane currents were recorded by using an Axopatch 200B amplifier. Data were sampled with a Digidata-1440 interface. The resting membrane potential was between −60 and −70 mV.  Table 4 shows electrical properties of recorded CA3 neurons.

The detection of the spontaneous excitatory currents was accomplished by using an automated routine based on the derivatives of the recording waveform with an algorithm similar to that previously published with a detection threshold of 10 pA compared to a noise level of about 2 pA [17]. Miniature and EPSCs recordings were performed at the reversal potential for GABAergic IPSCs (−70 mV).

Mossy fiber axons were electrically stimulated with a bipolar electrode (glass pipette with tip diameter of ~4 *μ*m filled with media solution, glued to a fine tungsten rod) placed in* stratum lucidum* (30–50 *μ*m from the edge of the pyramidal cell layer) at a lateral distance of 75–200 *μ*m from the recording pipette. The minimal stimulation intensity and duration were adjusted to observe failures of synaptic transmission (stimulus intensity and duration ranged between 140 and 160 microampere and 20 and 30 *μ*sec, Figure 4(b)).

In some experiments, a stimulating electrode was placed in* stratum radiatum* (150–200 *μ*m from* stratum pyramidale*) to activate CA3-CA3 synapses. Test pulses were delivered every 10 seconds; a hyperpolarizing current pulse (20 pA, 300 ms) was injected into the cell between test pulses to monitor input resistance and series resistance throughout each experiment. Accepted deviations from these parameters over the time-windows used for statistical analysis were less than 10%. Patch pipettes were pulled from borosilicate capillaries (Hingelberg, Malsfeld, Germany) and had 5–8 MΩ resistance before a seal was formed. The filling solution contained (in mM) the following: potassium gluconate 126, NaCl 4, MgSO_4_ 1, CaCl_2_ 0.02, BAPTA 0.1, glucose 15, ATP 3, HEPES 5, and GTP 0.1 and 1 mM picrotoxin (pH was adjusted to 7.2 with KOH). After obtaining a whole-cell configuration we waited 5 minutes before starting the recordings, in order to inhibit all IPSCs by picrotoxin.

At the end of the experiments we perfused in the bath 2 *μ*M DCG-IV, a receptor agonist of Group II metabotropic glutamate that blocks glutamate release from mossy fibers terminals.

Experimental traces were analyzed by using P-Clamp (Axon Instruments, Foster City, CA, USA) and Origin (Microcal Software, Northampton, MA, USA) software.

### 2.7. Statistics

After using Bartlett's test [17] for Homogeneity of Variances, two-way ANOVA repeated measures were used, where parameters reported in Figures 2(b) and 2(c) (number of approaches or total duration of approaches or average duration of approaches or latency of first approaches) were the dependent variable, the within subjects factor was “familiar object” or “novel object,” and the between subjects factor was “dextrin” or “*Hericium*” treatment. For emergence test a one-way ANOVA test was used.

Descriptive statistics, expressed as data, are reported as means ± standard error of the mean (SEM), and statistical comparisons in electrophysiological experiments analysis were made by using Student's *t*-test. Before applying the Student *t*-test, a QQ plot was generated and the Shapiro-Wilk test was performed for each pool of data to confirm a normal distribution.

In figures symbols indicate ^*∗*^*P* < 0.05, ^*∗∗*^*P* < 0.01, ^*∗∗∗*^*P* < 0.001.

More details about each statistical analysis are given in Section 3 and figures captions.

## 3. Results

### 3.1. Behavioural Tests

#### 3.1.1. Hr Supplementation Increases Novelty Exploration Behaviour of a New Environment

**Figure 1:**
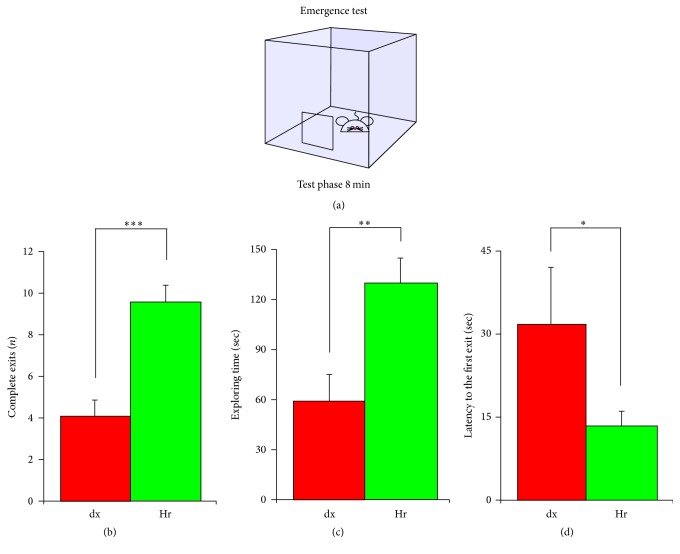
*H. erinaceus* increases novelty exploration behaviour. Emergence test in dx (*n* = 12) and Hr-dietary supplemented mice (*n* = 22) during an 8 min session. (a) Schematic of the experimental set-up and procedure used. Histograms show (b) the number of complete exits; (c) duration of exploring time; and (d) the latency of the first exit.

We first investigated the effect of oral supplementation with* H. erinaceus* on novelty exploration behaviour in healthy mice by using the emergence test (Figure 1(a)). We tested 18 dx mice and 22 Hr mice. The results show that the Hr mice had a higher frequency of complete exits (9.5 ± 0.8 versus 4.1 ± 0.8; *T* test *P* < 0.001, Figure 1(b)), spent more time out of the cage exploring the new environment (129.6 sec ± 14 versus 59.2 sec ± 15.8; *T* test *P* < 0.01, Figure 1(c)), and had a lower latency before the first exit (13.1 ± 2.6 sec versus 31.7 ± 10.3 sec; *T* test *P* < 0.05, Figure 1(d)). Overall, the number of complete exits (2.3-fold increase), the exploring time (2.2 times longer), and the decreased latency to the first exit (2.4 times shorter) all of them indicate an increase in novelty-seeking behaviour after the oral supplementation with* H. erinaceus* for two months.

#### 3.1.2. Hr Supplementation Increases Recognition Memory and Exploration of a Novel Object

**Figure 2:**
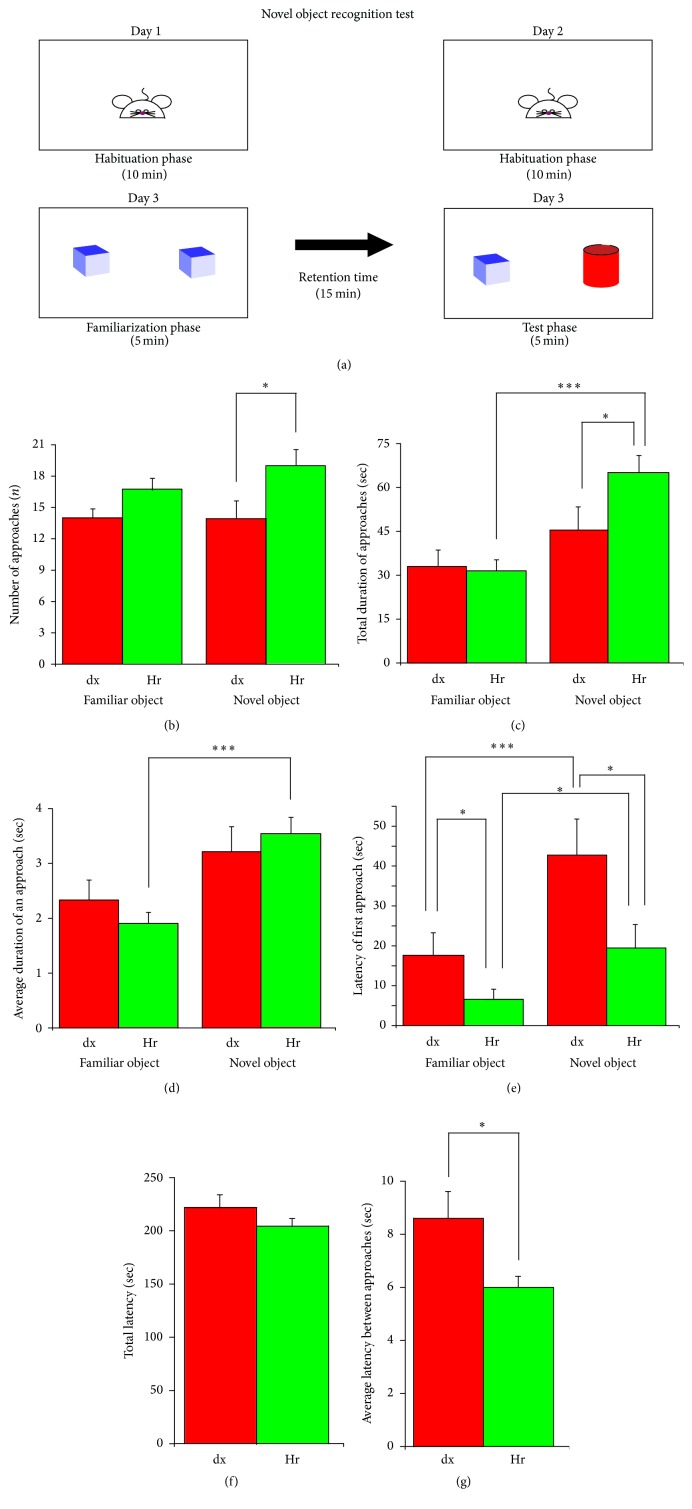
*H. erinaceus* increases recognition memory. Novel object recognition test (NOR) in dx (*n* = 10) and Hr-dietary supplemented mice (*n* = 15) during a 10 min session. (a) Schematic of the experimental set-up and procedure used. Histograms show (b) the number of approaches to the familiar and novel objects; (c) the total duration of approaches; (d) the average duration of an approach; (e) the latency to the first approach; (f) total latency; and (g) average latency between approaches.

Previous studies showed that* H. erinaceus* oral supplementation in mice with learning and memory deficits induced by intracerebroventricular administration of A *β*(25–35) amyloid *β*(25–35) peptide prevented cognitive deficits in a memory recognition task but not in a spatial working task [3]. Based on these findings, we decided to test the effect of* H. erinaceus* on recognition memory in wild-type mice by using the NOR task (Figure 2).

In the NOR task, in dextrin mouse (*n* = 12) the animals' natural inquisitiveness regarding the presence of a new object in the arena is expressed by a longer latency of the first approach to the novel object (42.7 ± 9 sec versus 17.6 ± 5.7 sec; *F*_3,46_ = 6.47, *P* < 0.001, Figure 2(e)). The number (13.9 ± 1.7 versus 14 ± 0.8, Figure 2(b)) and the total duration of approaches (45.4 ± 7.9 sec versus 33 ± 5.6 sec, Figure 2(c)) and consequently the average duration of an approach to the novel object did not reach the significance (3.2 ± 0.5 sec versus 2.3 ± 0.4 sec, Figure 2(d)).

When we compared the behaviour of dx and Hr mice (*n* = 19) toward the familiar object (Figures 2(b), 2(c), 2(d), and 2(e)), the only parameter that was statistically significantly lower in the latter was the latency for the first approach (Figure 2(e), 17.6 ± 5.7 sec versus 6.7 ± 2.4 sec, resp., *P* < 0.05), indicating that Hr and dx mice approach a familiar object in the arena in fairly similar ways.

Interestingly, the comparison of the behaviour of dx and Hr mice toward the novel object revealed that the number of approaches (13.9 ± 1.7 versus 18.9 ± 1.5; *F*_3,46_ = 3.24, *P* < 0.05, Figure 2(b)) and the total duration of approaches (45.4 ± 7.9 sec versus 65.4 ± 5.7 sec; *F*_3,46_ = 8.86, *P* < 0.05, Figure 2(c)) were significantly different, with the Hr mice showing 36% and 44% increases, respectively, in exploratory behaviour toward the novel object. Furthermore, the latency of the first approach (42.7 ± 9 sec versus 19.5 ± 5.9 sec; *F*_3,46_ = 6.47, *P* < 0.05, Figure 2(e)) and the average latency between approaches (8.6 ± 1 sec versus 5.9 ± 0.4, *T* test, *P* < 0.05, Figure 2(g)) were significantly lower (54% and 31.4% decrease) in Hr mice. However, neither the average duration of an approach (3.2 ± 0.5 sec versus 3.5 ± 0.3 sec, Figure 2(d)) nor the total latency (221.6 ± 12 sec versus 203 ± 7 sec, Figure 2(f)) reached significance. Briefly, Hr mice showed increased numbers and total duration of approaches when exploring the novel object in the arena and are less distrustful when approaching a novel object for the first time.

As expected, three out of four parameters that we measured in the behaviour of Hr mice with the familiar and novel objects were significantly different, with the total duration of approaches (31.5 ± 3.6 sec versus 65.4 ± 5.7 sec; *F*_3,46_ = 8.86, *P* < 0.001, Figure 2(c)), the average duration of an approach (1.9 ± 0.2 sec versus 3.5 ± 0.3 sec; *F*_3,46_ = 8.86, *P* < 0.001, Figure 2(d)), and the latency of the first approach (6.6 ± 2.4 sec versus 19.5 ± 5.9 sec; *F*_3,46_ = 6.47, *P* < 0.05, Figure 2(e)) all being significantly higher. We conclude that, after* H. erinaceus* dietary supplementation, mice spent more time exploring the novel object and increased the latency of the first approach compared with the familiar object.

To evaluate the discrimination between novel and familiar objects in dx and Hr mice, we calculated the mean novelty discrimination index (NI) by using the following formula: NI = (*n* − *f*)/(*n* + *f*) [18], where *n* is the average time with the novel object, and f is the average time with the familiar object. This index ranges from −1 to 1, where −1 means complete preference for the familiar object, 0 means no preference, and 1 means complete preference for the novel object. The NI index was 0.15 for the dx mice and 0.35 for the Hr mice.

In conclusion, Hr mice displayed very different behaviour compared with dx mice specifically when exploring novel objects, not familiar objects. The data are concordant and indicate that Hr mice show increased recognition memory performance.

### 3.2. Electrophysiological Data

**Figure 3:**
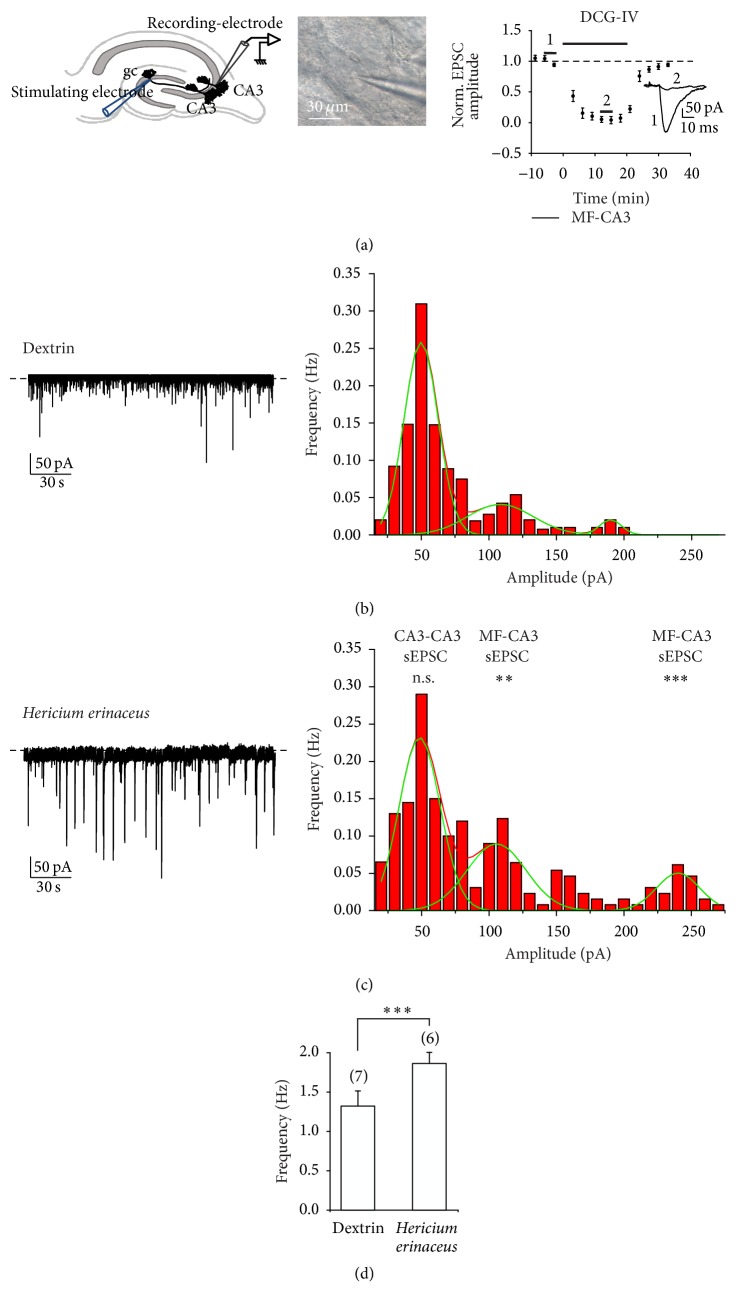
Spontaneous synaptic excitatory currents (sEPSCs) recorded from CA3 pyramidal cells. (a) Left, schematic drawing of experimental set-up. gc: granule cell, CA3 pyramidal cell. Middle, patch-pipette in a CA3 neuron. Right, bath-application of 2 *μ*M DCG-IV, a receptor agonist of Group II metabotropic glutamate that blocks glutamate release from mossy fibers terminals, selectively decreases mossy fiber response (see inset). ((b) and (c)) Excitatory currents (sEPSCs) and (d) frequency histogram of spontaneous activity recorded from CA3 pyramidal cells in dextrin treated mice (Dextrin, *n* = 6) and in* Hericium *supplemented mice (*Hericium erinaceus*, *n* = 5).

Pyramidal cells in hippocampal area CA3 receive both excitatory and inhibitory inputs. We recorded spontaneous and evoked synaptic currents in single CA3 pyramidal cells, in which GABAA receptor mediated responses were reduced with 1 mM intracellular picrotoxin (Figure 3(a)) and cells were voltage-clamped at −70 mV. In this experimental condition only excitatory currents are detected. CA3 pyramidal cells received excitatory synaptic inputs mainly from two sources: collateral axons from other CA3 pyramidal cells synapse on the medial apical and basal dendrites (CA3-CA3 synapse), and mossy fiber axons of dentate granule cells on the proximal, basal, and apical dendrite (mf-CA3 synapse).

#### 3.2.1. Hr Supplementation Increases the Frequency and Amplitude of Spontaneous Synaptic Excitatory Currents of Mossy Fiber-CA3 Synapse

As a direct measurement of the synaptic activity received by the CA3 neuron, we recorded spontaneous excitatory postsynaptic currents (sEPSCs) in single hippocampal CA3 pyramidal neurons voltage-clamped at −70 mV in mice after two months of oral supply with dextrin or* H. erinaceus *(Figure 3(a)).

By a multimodal fitting of the frequency histogram of spontaneous excitatory currents we can distinguish between collateral-CA3 and mossy fiber-CA3 events [19].

Figures 3(b) and 3(c) show representative traces (left) and frequency histogram (right) of spontaneous postsynaptic currents recorded in dx (*n* = 6 cells, Figure 3(b)) and Hr mice (*n* = 5 cells, Figure 3(c)). The frequency histograms obtained by representative neurons can be fitted by a trimodal Gaussian distribution with data binned into 10 pA bins (Figures 3(b) and 3(c), right). In our experimental condition the first Gaussian peak could be due to the multiquantal release of the recurrent transmission CA3-CA3. The frequency of the events contributing to the first peak is not statistically different between dx and Hr mice (0.32 ± 0.07 Hz for dx versus 0.28 ± 0.09 Hz for Hr).

Giant spontaneous EPSCs at the hippocampal mossy fiber to CA3 pyramidal cell are monoquantal [19–22] and in our experimental condition should correspond to the second Gaussian peak, at about 120 pA. The frequency of the spontaneous events contributing to the second peak is statistically higher in Hr mice compared to dx mice (0.13 ± 0.03 Hz for Hr versus 0.05 ± 0.01 Hz in dx; *T* test *P* < 0.01, Figure 3(c) compared to Figure 3(b), right). The third peak of the histogram at about 200–250 pA (Figure 3(c) compared to Figure 3(b), right) is only just sketched in dx mice, while it is clearly visible in Hr mice. This peak could be due to the mossy fiber-CA3 synapses originating from the younger granule cells that, as reported in literature, display an increase of the input resistance giving a dramatic increase in granule cells excitability ([23] see discussion). The overall spontaneous activity is significantly higher in Hr as compared to dx mice (Figure 3(d), 1.32 ± 0.19 Hz in dx versus 1.79 ± 0.26 Hz in Hr; *T* test *P* < 0.001).

In conclusion, spontaneous activity at hippocampal mossy fiber to CA3 pyramidal cell is significantly higher in Hr as compared to dx mice.

#### 3.2.2. Hr Supplementation Increases the Amplitude and Decreases the Failures Rate in Evoked Excitatory mf-CA3 Currents

By stimulating mossy fiber in the dentate gyrus (see  Materials and Methods), we recorded evoked mf-CA3 synaptic excitatory currents (EPSCs). Minimal mossy fiber stimulation was applied at low frequency (0.1 Hz). Two key inclusion criteria were stated for differentiating EPSCs evoked by mossy fibers and CA3 recurrent fibers: the latency of evoked responses is longer when stimulating mossy fibers (*n* = 16 cells, 6.4 ± 0.8 ms; *T* test *P* < 0.001) versus CA3 recurrent fibers (*n* = 16 cells, 2.1 ± 0.3 ms; *T* test *P* < 0.001) [24] and the inhibition by DCG-IV, an agonist of the metabotropic glutamate receptor 2. At the end of the experiments we blocked the mf-CA3 neurotransmission by local perfusion of DCG-IV (2 *μ*M, [25], see Figure 3(c) plot on the right).

**Figure 4:**
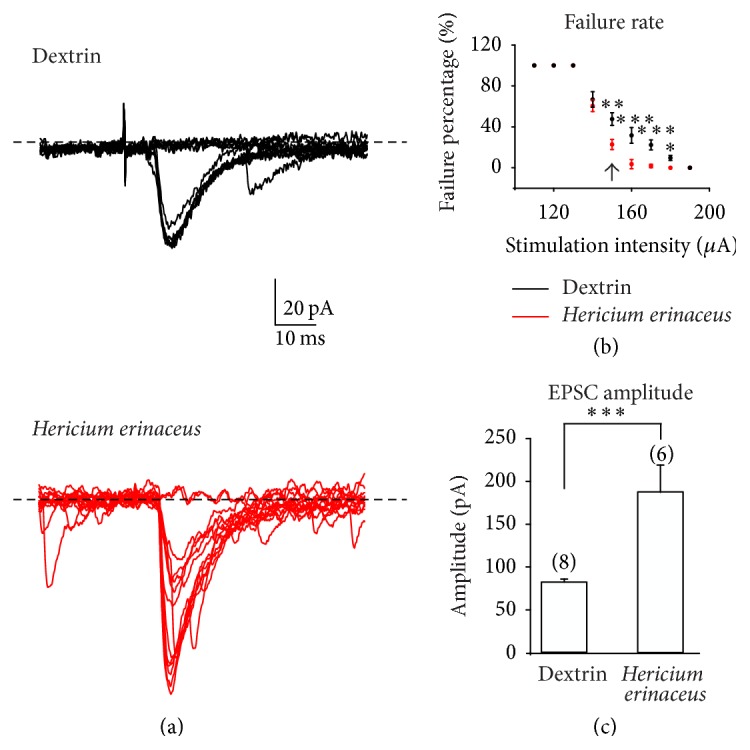
Mossy fiber-CA3 synapse evoked synaptic excitatory current (EPSCs) recorded after minimal stimulation at low frequency (0.1 Hz). (a) Experimental traces of evoked synaptic excitatory currents recorded in CA3 pyramidal cells for dextrin treated mice (top, Dextrin *n* = 8) and* H. erinaceus* oral supplemented mice (bottom,* Hericium erinaceus n* = 6). (b) Experimental design: mossy fibers were stimulated at different intensities (15 stimulations for each point) and the percentage of failure was calculated as the number of sweeps where no EPSC was evoked over the total number of stimulations per intensity point. The arrow inside the plot indicates the intensity point at which the representative traces in (a) are taken. (c) EPSCs amplitude histogram in dextrin treated mice (Dextrin) and in* H. erinaceus* oral supplemented mice (*Hericium erinaceus*).

EPSCs evoked by minimal mossy fiber stimulation were studied in the failures rate and in the amplitude (see representative traces in Figure 4(a), upper traces in dx mice and lower traces in Hr mice). We measured the failures percentage at minimal mossy fiber stimulation (see Materials and Methods). In dx mice (*n* = 8 cells) the failure percentage is significantly higher than in Hr mice (*n* = 8 cells, Figure 4(b), *P* < 0.001). The EPSC mean peak amplitude is significantly lower in dx mice compared to Hr mice (85.9 ± 17.9 pA versus 187.9 ± 68 pA, Figure 4(a); *T* test *P* < 0.001).* H. erinaceus* oral supply causes a significant decrease in percentage of failures and an increase in EPSCs amplitude.

#### 3.2.3. Hr Supplementation Increases Neurotransmitter Release in mf-CA3 Synapse

**Figure 5:**
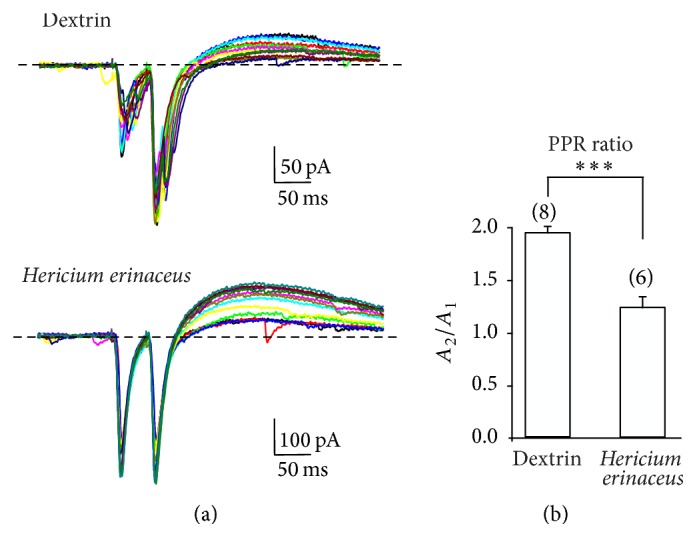
Paired-pulse stimulation: mossy fiber-CA3 synapse was stimulated by two pulses (interpulse interval 50 ms). (a) Experimental traces recorded in paired-pulse protocol for dextrin treated mouse (Dextrin, *n* = 8) and* H. erinaceus* supplemented mouse (*Hericium erinaceus*, *n* = 6). (b) Histograms of peak amplitude of the ratio between the second peak (*A*_2_) and the first peak (*A*_1_) for dextrin and Hr mice.

Paired-pulse facilitation (PPF) is a relatively simple experimental protocol to estimate the dynamic of presynaptic release during evoked neurotransmission [26]. PPF of the excitatory synaptic transmission at mossy fiber-CA3 synapse in the hippocampus was studied. While stimulating mossy fibers at low frequency (0.1 Hz), EPSCs were elicited in pairs with an interpulse interval of 50 ms between the two stimuli in both dx and Hr mice (Figure 5(a), representative traces in dextrin and* H. erinaceus*).

Mossy fiber-CA3 synapse always results in paired-pulse facilitation as described [27] and as confirmed in our dx mice (Figure 5(a), upper traces). In dx mice the ratio between the 2nd and 1st EPSP in a pair was 1.9 ± 0.3 (*n* = 8 cells, Figure 5(b)), indicating an increase in the release probability in the second of the two stimuli applied. In Hr mice, the ratio between the 2nd and 1st EPSP was 1.2 ± 0.2 (Figure 5(b), *n* = 6 cells) showing a significant reduction (*T* test *P* < 0.001).

If the neurotransmission is increased the paired-pulse ratio (PPR, i.e., the ratio between the 2nd and 1st EPSP in a pair) should decrease in the 2nd peak for the lower availability of neurotransmitter vesicle [28] and/or for the postsynaptic receptor desensitization [29].

A decrease in paired-pulse ratio, a decrease in failure rate, and an increase in EPSCs amplitude are concordant data indicating an increase in neurotransmission between mossy fiber and CA3 hippocampal neuron probably due to an increase in neurotransmitter release from mossy fiber axon.

## 4. Discussion

Our in vivo data concordantly demonstrate that wild-type mice supplemented with* H. erinaceus *increased their exploration of novel stimuli. It has been recognized that several parameters of a task need to be recorded to support the validity and interpretation of the data of a behavioural experiment [30]. In our emergence test, the decreased latency of the first exit, the increase in the frequency of complete exits, and the longer duration of exploring time offer convergent evidences of increased novelty-seeking behaviour.

Recognition memory, a form of declarative memory, can generally be defined as the ability to discriminate the novelty or familiarity of previous experiences by identifying when something (e.g., an object or an environment) has already been encountered. The results of the NOR task indicate that the Hr mice spent more time approaching the novel object than the familiar object; the decreased latency of the first approach and the increase in the frequency of approaches, combined with the longer duration of approaches, further support a state of increased novelty exploration behaviour. Conversely, the NOR test revealed no differences between Hr and dx mice in the exploration of the familiar object, indicating that the increase in exploratory activity is specifically oriented to the novel object.

The ability to cope with novelty is essential in all mammal species [31]. Novelty-seeking has been identified as one of the six major human personality dimensions, whereas neophobia describes hesitancy to engage with novel objects and places and can be considered a risk factor for anxiety disorders [32, 33]. A low level of exploratory activity towards novelty is interpreted as a sign of anxiety-like behaviour, whereas a high level reflects less anxiety [34]. Furthermore, reduced novelty-seeking and, in turn, increased neophobia can be considered core symptoms of depression; these behaviours are closely related to rigid evaluative patterns and reduced coping flexibility that also characterize the depressive state [35–38]. A recently published paper described the reduction of depression and anxiety by 4 weeks' intake of* H. erinaceus* dietary supplementation in 30 female subjects [5].

As we did, several studies have used the novelty approach as a measure of anxiety and the parameters measured were the latency, the frequency, and the duration of approaches [39, 40].

When rodents explore novel objects the pathway from the perirhinal cortex to lateral entorhinal cortex and then to the dentate gyrus and CA3 is engaged, whereas the pathway from the perirhinal cortex to lateral entorhinal cortex and then to CA1 is involved when familiar object s are explored [41].

Therefore, we focused our attention in this pathway, in particular on the final projection from the dentate gyrus (the mossy fiber tract) to the CA3 area (the pyramidal CA3 neurons).

The differential effect in Hr for novel versus familiar objects is reflected in the network as an increased neurotransmission at the mossy fiber-CA3 activity on both spontaneous (amplitude and frequency) and evoked glutamatergic events (amplitude).

The electrophysiological data obtained by recording in CA3 pyramidal neurons show some substantial differences in a number of features on both spontaneous excitatory postsynaptic currents (sEPSCs) and evoked excitatory postsynaptic currents (EPSCs) in mf-CA3 synapse in mice treated with* H. erinaceus* compared to those treated with dextrin.

In Hr mice, sEPSCs recorded from mf-CA3 neurons are increased in frequency and amplitude while a nonstatistical change in CA3-CA3 spontaneous currents was observed. In vivo and in vitro it was demonstrated that only a 2–5% fraction of granule cells in the hippocampal dentate gyrus neurons are spontaneously active and a monoquantal release was described [42–44].

Again, in Hr mice evoked mf-CA3 EPSCs are higher in amplitude and the frequency of the failures is lower. Furthermore, in paired-pulse protocol, we revealed a decrease in paired-pulse facilitation in EPSCs recorded in Hr mice. All these data together are in agreement with an increase in neurotransmitter release by the mossy fiber axons.

Adult hippocampal neurogenesis is the most interesting of the neurogenic zones in the adult brain, because it is involved in higher cognitive function, most notably memory processes, and certain affective behaviour [45]. In particular, in the dentate gyrus, adult and persistent hippocampal neurogenesis generates new excitatory granule cells in the dentate gyrus and contributes significantly to plasticity across the life span.

The hypothesis of on increased neurogenesis is in agreement with our data. As extensively reported by both in vitro and in vivo investigations [24] the newborn granule cells (GCs) have peculiar electrophysiological properties. Among them the most relevant one consists of a higher input resistance [46, 47] and of a lower rheobase [48]. Both of these parameters lower the threshold for the generation of the action potential in the newborn compared to the mature GCs, so increasing the neuron excitability. Consequently, a lower threshold for the generation of the action potential increases the likelihood that an action potential is triggered from the newborn GCs with two major consequences: an increase in the frequency of spontaneous events and a decrease in the frequency of the failures in evoked currents recorded in the postsynaptic CA3 pyramidal cells.

Moreover, while the mature GCs spontaneous firing activity is normally characterized by a single spike, the new born GCs deliver a burst of action potentials at high frequency [48]. Consequently, due to the temporal summation, a higher number of action potentials will provide a larger amount of the neurotransmitter released for every event and this will be detected as an increase in the amplitude of the excitatory spontaneous and evoked postsynaptic currents. Briefly, even though more experiments must be performed in future, the findings described in our investigation support the hypothesis of an increased neurogenesis in the hippocampal dentate gyrus as a consequence of* H. erinaceus *dietary supplementation in wild-type animals.

Hippocampal neurogenesis would impact the animal behaviour. A neurogenetic hypothesis of depression was originally formulated upon the demonstration that neurogenesis is regulated negatively by stressful experiences and positively by treatment with antidepressant. Since then much work has established that newborn neurons in the dentate gyrus are required for mediating some of the beneficial effects of antidepressant treatment [49–51].

What might be the molecular mechanism at the base of all those effects mediated by* H. erinaceus*?

Interestingly it was demonstrated that chronic intracerebroventricular infusion of nerve growth factor improves recognition memory in the rat [52] through an increase in the expression of NGF and of its receptor, TrkA, and of the synaptic vesicle protein, synapsin. This effect was paralleled by an increase in cell proliferation in the dentate gyrus of NGF treated rats. These data indicate that NGF chronic infusion can stimulate an improvement in learning and memory that is associated with specific cellular changes in the hippocampus, including synaptogenesis and cell proliferation.

Overall, our data show that* H. erinaceus* supplementation for two months has influential effects on wild-type mice, increasing glutamatergic synaptic drive novelty exploration behaviour and recognition memory in hippocampus.

Our study on the effects in wild-type mice of* H. erinaceus* oral supplementation on brain in both in vivo and in vitro experiments yielded several key findings that we hope will pave the way for new studies in healthy humans and bridge the gap between the millenary Eastern medicine and our Western medicine [53–55].

## Figures and Tables

**Table 1:** Nutrient composition of dietary supplements (supplied by A. V. D. Reform, Noceto, Parma, Italy).

Components	Per capsule (mg)
*Hericium *mycelium	400

*Hericium* fruiting bodies extract	100
Titled in polysaccharides	38.6

**Table 2:** Nutritional composition of *H. erinaceus* extract.

Analyte	Result	Unit
Calorie	2.23	Kcal/g
Crude proteins	8.25	%wt
Crude fat	0.17	%wt
Crude fiber	5.92	%wt
Polysaccharides/total glucan	>45	%wt
Sodium	0.0146	%wt

**Table 3:** Nutritional composition of *H. erinaceus* mycelium.

Analyte	Result	Unit
Calorie	1.98	Kcal/g
Crude proteins	10.22	%wt
Crude fat	1.02	%wt
Crude fiber	39.2	%wt
Polysaccharides/total glucan	>37	%wt
Sodium	0.0031	%wt

**Table 4:** Electrical properties of neurons in Dx mice (*n* = 16) and Hr mice (*n* = 16). *R*_in_, input resistance; *C*_*m*_, membrane capacitance; and *R*_*s*_, series resistance. Differences are not statistically significant.

	Dx mice	Hr mice
*R*_in_ (Mohm)	122 ± 22	115 ± 11
*C*_*m*_ (pF)	294 ± 17	211.8 ± 15.2
*R*_*s*_ (Mohm)	6.7 ± 1.7	5.7 ± 0.5
